# Nonstrabismic binocular dysfunctions and cervical complaints: The possibility of a cross-dysfunction

**DOI:** 10.1371/journal.pone.0209710

**Published:** 2019-01-15

**Authors:** María Carmen Sánchez-González, Verónica Pérez-Cabezas, Estanislao Gutiérrez-Sánchez, Carmen Ruiz-Molinero, Manuel Rebollo-Salas, José Jesús Jiménez-Rejano

**Affiliations:** 1 Department of Physics of Condensed Matter, Optics area, University of Seville, Seville, Spain; 2 Department of Nursing and Physiotherapy, University of Cadiz, Cadiz, Spain; 3 Department of Surgery, Ophthalmology area, University of Seville, Seville, Spain; 4 Department of Physiotherapy, University of Seville, Seville, Spain; Faculty of Medicine, Cairo University, EGYPT

## Abstract

The aim of this study is to establish a relationship between non-strabismic binocular dysfunction and neck pain. One hundred twelve participants underwent binocular vision assessment by evaluating horizontal heterophoria, horizontal and vertical fusional vergence ranges and vergence facility. The subjects were classified into two groups: binocular anomalies and normal binocular function. Neck complaints were measured with the Neck Disability Index, visual analogue scale, cervical range of motion, deep-flexor muscle activation score (AS) and performance index (PI). Our results showed that participants with low AS had significantly altered values of lateral phoria (near) (mean = -6.99 SD ± 6.96 PD) and PFV (near) blur (mean = 9.49 SD ± 5.45 PD) against those who presented normal AS (lateral phoria (near) mean = -3.64 SD ± 6.37 PD; PFV (near) blur mean = 12.84 SD ± 6.20 PD). In addition, participants with NFV (near) recovery outside the norm had a significantly lower right side-bending (mean = 35.63 SD ± 8.35 PD) than those within the standard (mean = 39.64 SD ± 9 PD). The subjects with binocular vision impairment showed a diminished response to the deep cervical musculature, with low AS and PI, as well as a tendency to suffer from cervicalgia of more than three months’ evolution and a lower range of motion.

## Introduction

The use of new technologies requires prolonged visual demand in a restricted visual space. This situation implies a continuous over-exertion of accommodation and vergence, which alter the efficiency of the visual system, and a diverse symptomatology appears that includes asthenopia and performance problems [1,2]. Accommodative dysfunctions and nonstrabismic binocular dysfunctions are frequent visual alterations arising from this situation [3,4]. Similarly, this situation increases musculoskeletal discomfort in the neck area so that both visual symptoms and muscular complaints coexist [5,6].

Different authors have reported the joint prevalence of visual and cervical symptoms. Zetterberg et al. [7,8] established relationships between highly demanding visual situations during near work and discomfort in the neck and shoulder. Domkin et al. [9] found that sustained contraction of the ciliary muscle was associated with an increase in trapezius muscle activation level, which may contribute to the development of musculoskeletal complaints in the neck area. Richter et al. [10] reported the coexistence of both symptoms in similar situations. Thus, cross-dysfunction between the two systems is a possibility.

There is a relationship between visual dysfunction and neck muscle alterations[7–13]. The musculoskeletal disturbances that occur in the neck have been analyzed simultaneously with visual functions by inserting monocularly and binocularly positive and negative lenses while the subject fixes a stimulus [7,8,10–13], or with the help of a photorefractor during the focusing of a moving target located at 40 cm [9]. In some works, the relationships between visual symptoms and neck pain were studied by using questionnaires that we considered to have a certain degree of subjectivity [5,14–16]. However, in no case did they measure each of the parameters that define binocular vision while looking for dysfunctions and their relationships with the existence of cervical diseases.

The visual parameters that are normally measured to determine the binocular vision status are the horizontal heterophoria value, the amplitude of both the positive (PFV) (convergence) and negative (NFV) (divergence) fusional vergences, range of vertical vergences (VV), vergence facility testing (VF) and near point of convergence (NPC) [17,18]. Other parameters, such as negative relative accommodation, positive relative accommodation, binocular accommodative flexibility, and accommodative convergence/accommodation (AC/A) ratio, evaluate the interaction between the vergence and accommodative systems and will not be specified in our study owing to the age of the subjects, since the value of these variables decreases with age [19]. For the same reason, the value of the (NPC) [20] is not specified.

The aim of our study is to determine if there is a relationship between suffering nonstrabismic binocular dysfunction and suffering neck pain. We also propose a complete and exhaustive assessment of the state of binocular function.

## Materials and methods

### Design

A descriptive, cross-sectional, correlational study, conducted from March 1, 2017 until December 31, 2017 at the Faculty of Pharmacy, at the Optics and Optometry Titling facilities, Faculty of Pharmacy, University of Seville, was performed.

### Ethics

The research followed the tenets of the Declaration of Helsinki; informed consent was obtained from the subjects after explaining the nature and possible consequences of the study; and the Institutional Review Board of the University Hospital Virgen Macarena of the University of Seville approved the research.

### Subjects

The selected population was made up of students, professors and administrative and service personnel of the University of Seville. The proposal for participation in the present study was sent via email to the entire university community of the Faculty of Pharmacy at the University of Seville. Those interested totaled 143 subjects. All subjects were informed about the study verbally and in writing. Once informed in depth, 6 people refused to participate, and 3 did not sign the informed consent, leaving a total of 134 participants who gave their consent to participate in this research.

All subjects had at least 20/20 best-corrected visual acuity and an absence of ocular motility defects, strabismus, nystagmus, corneal ectasias, suppression, diplopia, amblyopia, and any ocular or systemic disease that could affect the results. Subjects who had undergone some type of ocular surgery or had a history of head trauma, cervical fracture or surgery in this area; persons with intellectual disabilities or any problems that prevented them from completing the Neck Disability Index (NDI); or who suffered any type of degenerative disease or neurological alteration were excluded. Twenty-two out of a total of 134 potentially eligible subjects were excluded [corneal ectasias (n = 4); suppression (n = 3); diplopia (n = 1); nystagmus (n = 3); amblyopia (n = 6); refractive surgery (n = 5)]. The sample consisted of 112 subjects with a mean age of 39.8 years [standard deviation (SD) ± 14.97 probability distribution (PD)], aged from 18.0 to 65.0 years, and comprised 61 (54.5%) women and 51(45.5%) men.

### Measurements

The measurements used in our study by physiotherapists were:

- The cervical joint range measured with the cervical range of motion (CROM) instrument [21], in degrees (°) [flexion, extension, right (RSB) and left side-bending (LSB) and right and left rotation (RR, LR)].
- The condition of the deep flexor musculature [activation score (AS) and performance index (PI)], using the craniocervical flexion test (CCFT), was assessed with the ChattanoogaTM stabilizer pressure biofeedback device (Chattanooga Stabilizer Group Inc., Hixson, TN) [22].
- The cervical disability assessed with the NDI questionnaire (range 0–50) [23].
- Neck pain intensity was evaluated with the visual analogue scale (VAS), range 0–10.
- Cervicalgia evolution [24,25] of three or more months was assessed with a qualitative nominal dichotomous variable, presenting two categories, "Yes/No".

The variables with which the binocular function was measured were:

- The magnitude of the horizontal heterophoria (Prism Diopters, Δ), was measured at distance and near with an occluder, a prism bar, and an accommodative target.
- The amplitude of both the positive (convergence) and negative fusional vergences (divergence), were measured using the rotary prisms of the phoropter (ESSILOR MPH100E S / N 000104 phoropter).
- Vertical fusional vergences (VV, Δ) were measured using the rotary prisms of the phoropter, (ESSILOR MPH100E S / N 000104 phoropter).
- Vergence facility (VF) (cycles per minute, cpm) was quantified with a prismatic combination 3 Δ base-in (BI) /12 Δ base-out (BO).

A new variable was defined from the values of the previous variables: Sheard’s criterion [26], which describes the global state of the binocular vision in individuals with normal and outside-normal binocular vision.

### Procedures followed

#### Physiotherapy assessment (neck complaints)

A trained physiotherapist evaluator, with seven years of experience in these types of measurements, assessed in this order: 1) the patient’s pain using the VAS and cervical disability using the NDI questionnaire before beginning the other evaluations; 2) cervical range of motion while sitting: the participant was asked to actively perform flexion, extension, right side-bending (RSB), left side-bending (LSB), right rotation (RR) and left rotation (LR) movements three times each to find the mean of the measurements; and 3) the activity of the deep flexor musculature with the CCFT (AS and PI). The CCFT was performed with the participant in a supine position with the neck in a neutral position (without a pillow). The device was positioned under the neck and against the occiput. It was inflated, once placed, to the 20 mmHg level. The patient moved the head as if they were saying "Yes". A trained examiner observed and corrected any substitution of movements. Everyone was instructed to perform craniocervical flexion of the neck at five pressure levels (22, 24, 26, 28 and 30 mmHg), and hold the position firmly. If they achieved this, they had to relax the muscles, and then repeated the movement for each position (obtaining the "activation score" (AS), depending on the pressure, with a range of 1 to 5). When the AS was established, the therapist asked them to maintain the pressure, with minimal superficial muscle activity, performing 10 sustained 10-second repetitions. The number of repetitions was called "performance." A performance index (PI) was calculated by multiplying the AS by the performance. Neck pain case studies using this test showed that the scores were less than 4 in AS and less than 10 in PI in patients with cervical disorders. These subjects presented neuromotor control with a deteriorated activation of the deep cervical flexor muscles. This deterioration seems generic to cervical pain disorders [22,24].

All data were collected on a record sheet by another physiotherapist. At the time of data collection, the assessors did not know the level of discomfort of the participants. This was established after the data processing. The physiotherapists were blinded regarding the optometric evaluation and vice versa.

#### Optometry assessment (binocular dysfunctions)

Once physiotherapy assessment was finished, and after a 60-minute break, patients were moved to an adjoining room where a licensed optometrist performed an optometric examination.

Horizontal heterophoria was quantified with alternate cover test (ACT), which occluded each eye alternatively for 5 seconds. At any moment, both eyes remained uncovered, so binocular vision was not allowed to adopt a rest position of the visual axes. At the same time, the prism bar was placed. The prismatic power was increased until the movement of the eyes was neutralized. This constituted the phoria value. Prisms BO neutralizes esophoria and prisms BI neutralizes exophoria [27]. The alternate cover test is considered the most significant diagnostic procedure and is the most commonly used objective clinical test to date [28].

The range of horizontal and vertical vergences was measured using Risley rotating prisms of the phoropter and taking three measurements spaced 15 seconds apart. Prisms were introduced at a rate of 1 Δ per second. The patient had to indicate when saw blurred text (blur point) or doubled (break point) in horizontal vergences. Double text indication (break point) was only in vertical vergences. The prismatic power was decreased until the patient merged the image again (recovery point) [29].

VF was measured by changing between BI and BO prisms (3 Δ BI / 12 Δ BO), requiring the subjects to converge and diverge. The fixation point was a near Snellen chart located 40 cm from the subject. We presented a VA equivalent of 0.8. The measurement involved introducing the BI first. The patient clarified the image. Next, we changed to BO. The process alternated for 1 minute. The number of complete cycles (one BI and one BO prism) was the value of the VF [30].

### Data analysis

The data were analyzed with the SPSS 24 package for Windows (SPSS Science, Chicago, United States). The normality of our variables was verified with the Shapiro-Wilk test. A descriptive data analysis was developed, showing the count and proportion of each category in the qualitative variables and the mean and SD or in its defect the median and the interquartile range (IQR), and the range (minimum-maximum) in the quantitative ones. Then, the relationships between the variables considered were studied by calculating the Pearson coefficient (r). Then, the values of disability, pain, mobility, AS and PI were compared in the groups in which we differentiated the subjects according to the normative values of variables that described the state of binocular vision. After this, the values of binocular vision were compared in the groups in which we differentiated the subjects according to the normative values of NDI, AS, PI and suffer cervicalgia of 3 or more months’ evolution. Finally, we analyzed if there was a relationship when comparing the categorized values (if they were inside or outside the norm) of the variables that defined the state of the vergence function relative to the normative levels of the variables that described the neck area. In all these analyses, Student t-test or Welch’s t-test was used, as required, and for the variables that were not normally distributed, the Mann-Whitney U-test was used. As a complement to the above analyses, the effect size was calculated to determine the value of the standardized difference of means (d of Cohen) when the t-test was performed, while we followed the criteria of Grissom [31–33] when using the Mann-Whitney test. When the relationship between categorized variables was studied, the Pearson chi-squared test was employed or, failing this, the Fisher exact test. All statistical tests were performed considering a 95% confidence interval (CI) (P <0.05). In addition, the differences that approached said statistical significance are shown.

## Results

Table 1 shows the mean values of the variables that defined the state of the binocular vision, as well as the classification of participants inside or outside the normative values of these variables. Table 2 shows the values of the variables related to range of motion, pain, neck disability, and the state of the deep flexor muscle activity.

**Table 1:** Characteristics of the variables that defined binocular vision.

Variable		n	Mean ± SD (PD)	Range	Classification of subjects according to normative values n (%)	
					Outside the norm	Inside the norm
Sheard’s Criterion	Distance	110	-	-	21 (19.1)	89 (80.9)
	Near	110	-	-	56 (50.9)	54 (49.1)
Lateral phoria, Δ	Distance	112	-0.52 ± 2.18	-12-8	15 (13.4)	97 (86.6)
	Near	112	-5.52 ± 6.88	-25-16	52 (46.4)	60 (53.6)
NFV Distance, Δ	Break	111	8.51 ± 2.28	4–14	18 (16.2)	93 (83.8)
	Recovery	111	4.50 ± 1.85	0–9	12 (10.8)	99 (89.2)
NFV Near, Δ	Blur	86	10.95 ± 4.56	4–30	39 (45.3)	47 (54.7)
	Break	112	17.11 ± 5.13	6–36	49 (43.8)	63 (56.2)
	Recovery	112	11.59 ± 4.67	0–20	29 (25.9)	83 (74.1)
PFV Distance, Δ	Blur	75	9.97 ± 4.51	2–24	23 (30.7)	52 (69.3)
	Break	107	16.29 ± 6.72	6–36	31 (29)	76 (71.0)
	Recovery	107	8.10 ± 4.58	0–27	31 (29)	76 (71.0)
PFV Near, Δ	Blur	82	11 ± 6	2–28	54 (65.9)	28 (34.1)
	Break	109	17.17 ± 7.46	6–38	64 (58.7)	45 (41.3)
	Recovery	109	9.74 ± 6.12	0–32	16 (14.7)	93 (85.3)
Vergence facility, cpm		90	9.49 ± 4.60	0.5–22	73 (81.1)	17 (18.9)
Vertical Vergence Distance, Δ	Break	112	3.18 ± 0.95	0–6	10 (8.9)	102 (91.1)
	Recovery	112	0.93 ± 0.78	0–3	36 (32.1)	76 (67.9)
Vertical Vergence Near, Δ	Break	112	3.54 ± 1.17	2–10	14 (12.5)	98 (87.5)
	Recovery	112	1.16 ± 0.85	0–3	26 (23.2)	86 (76.8)

SD = standard deviation. PD = probability distribution. Δ = prism diopters.

**Table 2:** Characteristics of variables that defined disability, range of motion, activation score, performance index and neck pain (n = 111).

Variable	Mean ± SD (PD)	Range	Classification of subjects according to normative values n (%)	
Cervicalgia 3 months	-	-	Yes	67 (60.4)
			No	44 (39.6)
Neck Disability Index, 0–50	6.37 ± 6.32	0–26	NDI < five	57 (51.4)
			NDI ≥ five	54 (48.6)
Activation Score of deep cervical musculature	4.43 ± 3.06	0–10	AS < four	47 (42.3)
			AS ≥ four	64 (57.7)
Performance Index of deep cervical musculature	10.84 +/- 12.71	0–70	PI < ten	39 (35.1)
			PI ≥ ten	72 (64.9)
Visual Analogue Scale, 0–10	2.67 +/- 2.78	0–8.80		
Flexion, °	50.48 ± 10.78	21.33–74.33		
Extension, °	60.79 ± 14.6	13.33–110		
Right Side-Bending, °	38.59± 8.98	18–60		
Left Side-Bending, °	42.94 ± 10.48	16–67.67		
Right Rotation, °	63.49 ± 10.77	38–96.67		
Left Rotation, °	67.15 ± 12.08	35.67–97.33		

SD = standard deviation. PD = probability distribution.

### Correlation between the variables that define the state of the cervical region and binocular vision

Significant relationships were found between: 1) Neck Disability Index (NDI) and VV (near) recovery (r = -0.189, p = 0.047); 2) activation score (AS) and PFV (near) blur (r = 0.248, p = 0.025); 3) performance index (PI) and lateral phoria (distance) (r = 0.209, p = 0.028), NFV (distance) recovery (r = -0.192, p = 0.044), PFV (near) blur (r = 0.233, p = 0.035), PFV (near) recovery (r = 0.195, p = 0.044); 4) flexion and PFV (near) blur (r = 0.284, p = 0.010); 5) extension and lateral phoria (near) (r = 0.205, p = 0.030), PFV (distance) recovery (r = 0.221, p = 0.023), and vergence facility (r = 0.213, p = 0.046); 6) right side-bending (RSB) and NFV (distance) break (r = 0.218, p = 0.022); 7) left side-bending (LSB) and lateral phoria (near) (r = 0.188, p = 0.048); 8) right rotation (RR) and lateral phoria (distance) (r = 0.211, p = 0.026), lateral phoria (near) (r = 0.228, p = 0.016) and PFV (near) blur (r = 0.272, p = 0.014); 9) left rotation (LR) and lateral phoria (near) (r = 0.212, p = 0.026), PFV (near) break (r = 0.293, p = 0.002), PFV (near) recovery (r = 0.243, p = 0.011) and VV (distance) recovery (r = 0.219, p = 0.021). Correlations at the descriptive level were nonsignificant between NDI and PFV (distance) recovery (r = -0.185, p = 0.058), between PFV (near) break and AS and PI (r = 0.174, p = 0.071 and r = 0.185, p = 0.055, respectively), and between LR and VV (near) recovery (r = 0.174, p = 0.067). In all cases the correlations found were small.

### Comparison of the state of the cervical region of subjects with normal binocular vision versus those outside the norm

**Fig 1:**
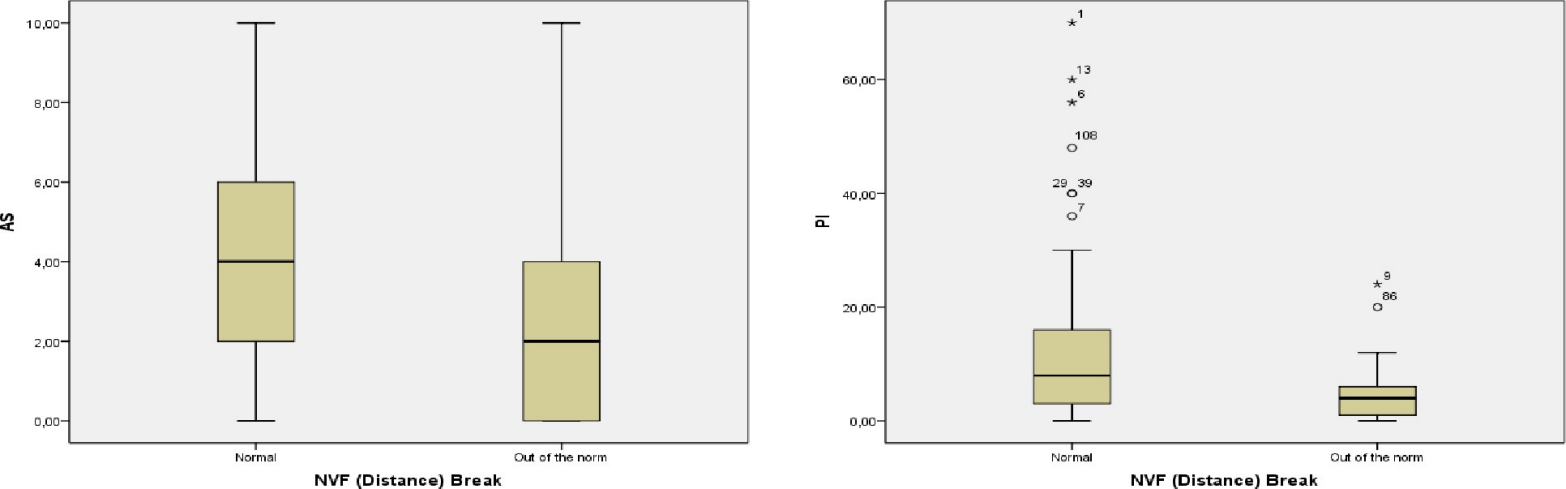
Differences in Activation Score (AS) and Performance Index (PI) in subjects with normal NFV (distance) Break or above said normal values.

Regarding the comparison of the values of the variables related to pain, and deep cervical musculature activity, in the subjects who presented a state of normal or outside-of-normal binocular vision, there were no significant differences in any case, except in the NFV (distance) break. Participants with NFV (distance) break values within the norm, which were 92, had an AS (median = 4; IQR = 2–6) and a PI (median = 8; IQR = 2.5–16) significantly higher (p = 0.026, effect size = 0.33 and p = 0.023, effect size = 0.34 respectively) than the remaining 18 subjects who had higher-than-normal NFV (distance) break (AS: median = 2; IQR = 0–4.5; PI: median = 4; IQR = 1–6.5). These results are shown graphically in Fig 1.

**Fig 2:**
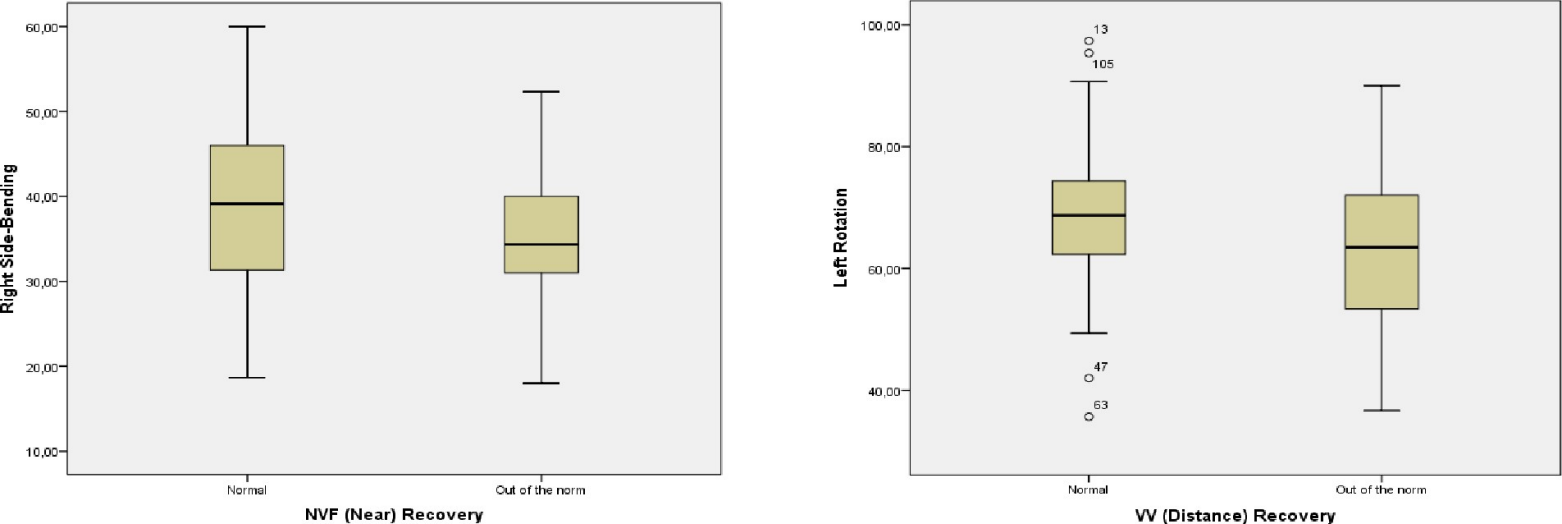
Normative values of Vergential Function versus cervical mobility. VV: vertical vergence.

Regarding the levels of the variables related to neck mobility in individuals with normal values, or outside the norm, Table 3 shows the differences found in the variables that defined the state of binocular vision, as does Fig 2.

**Table 3:** Comparison of the cervical mobility according to whether the subjects were inside the normative values, or not, in the variables related to the state of binocular vision (vergential function).

Variable		Extension, º	Right Side-Bending, º	Left Rotation, º
NFV (Near) Break (n = 111)	Satisfy the norm (n = 62), mean +/- SD(PD)	-	39.96 +/- 8.32	**-**
	Do not satisfy the norm (n = 42), mean +/- SD(PD)	-	36.87+/- 9.55	**-**
	**p-value**	-	0.072	**-**
NFV (Near) Recovery (n = 111)	Satify the norm (n = 82), mean +/- SD(PD)	60 (52.67–72.67)*	39.64 +/- 9	**-**
	Do not satisfy the norm (n = 29), mean +/- SD(PD)	56.67 (49.33–65.17)*	35.63 +/- 8.35	**-**
	**p-value**	0.074†	**0.038**	**-**
PFV (Distance) Break (n = 106)	Satisfy the norm (n = 75), mean +/- SD(PD)	62.82 +/- 14.25	-	**-**
	Do not satisfy the norm (n = 31), mean +/- SD(PD)	57.14 +/- 13.06	-	**-**
	**p-value**	0.059	-	**-**
Vertical Vergence (Distance) Recovery (n = 111)	Satisfy the norm (n = 75), Mean +/- SD(PD)	-	-	68.82 +/- 11.30
	Below the norm (n = 36), Mean +/- SD(PD)	-	-	63.68 +/- 13.04
	**p-value**	-	-	**0.035**
Vertical Vergence (Near) Recovery (n = 111)	Satisfy the norm (n = 85), mean +/- SD(PD)	-	-	68.36 +/- 12.07
	Below the norm (n = 26), Mean +/- SD(PD)	-	-	63.21 +/- 11.45
	**p-value**	-	-	0.056

SD = Standard Deviation. PD = Probability Distribution.

*Median and Interquartile range (IQR) are shown.

†Mann-Whitney *U* test was used.

### Comparison of the binocular vision status of subjects with cervical region alteration compared to those with normal values

**Fig 3:**
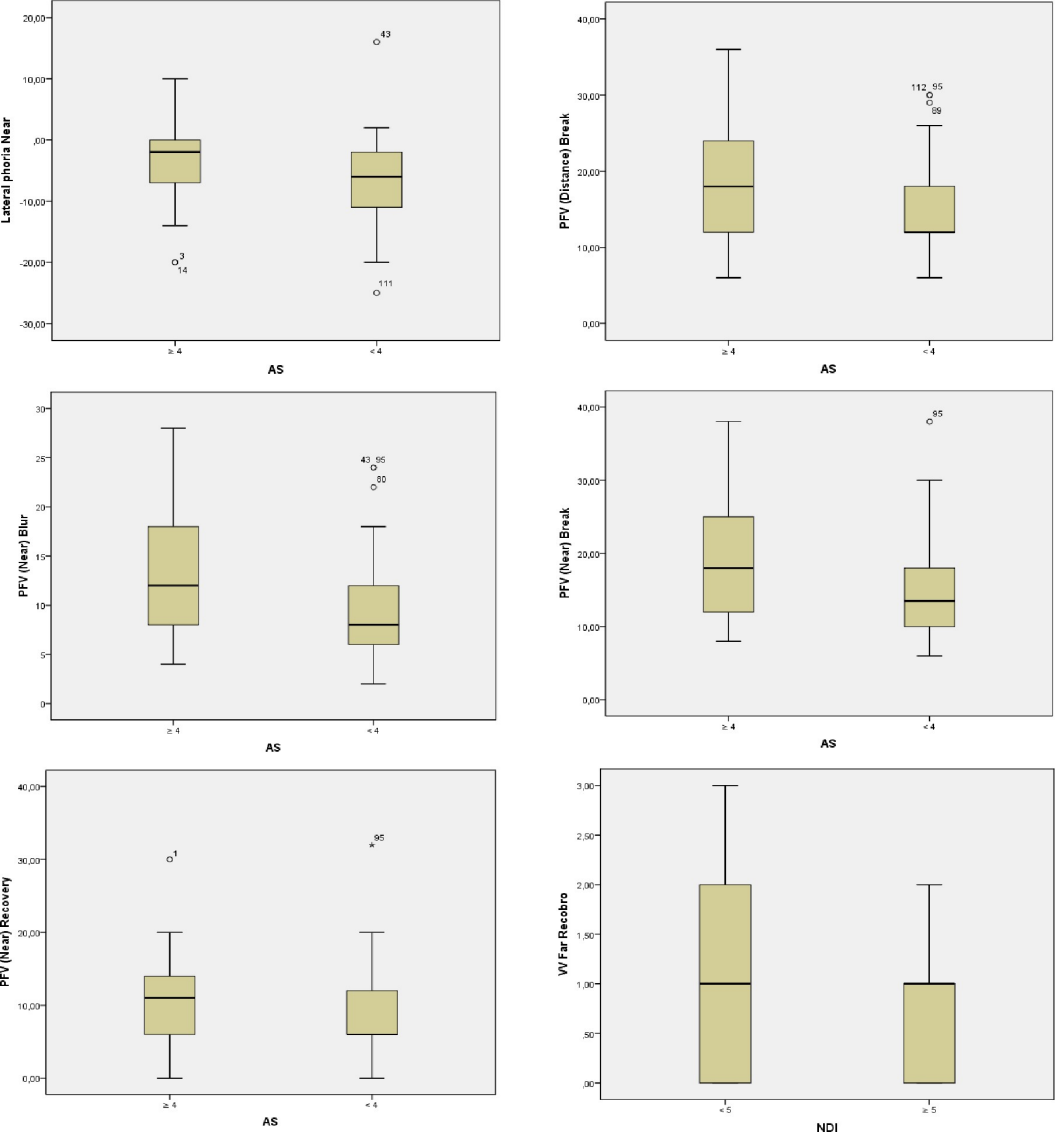
Activation Score (AS) and Neck Disability Index (NDI) normative values versus Vergential Function. VV: Vertical Vergence.

In the subjects who presented a state of normal or outside-of-normal AS, there were significant differences in the value of the variables that described the state of the binocular vision. These results are shown in Table 4 and graphically in Fig 3. On the other hand, when patients were classified according to the NDI value, in VV recovery (far), a significant difference was found (p = 0.049; effect size (Cohen’s d) = 0.38). This variable presented lower results (mean = 0.78 Δ SD ± 0.69PD Δ) in the 54 individuals who had a NDI greater than or equal to 5 points than in the 57 (mean = 1.07 Δ SD ± 0.84PD Δ) who had less than 5 points. These analyses are described in Fig 3.

**Table 4:** Comparison of the state of binocular vision in subjects with AS higher or the same as 4 points as opposed to those with AS lower than 4 points.

Variable	AS				
	≥ 4		< 4		p-value
	n	mean ± SD(PD)	n	mean ± SD(PD)	
Lateral phoria (Distance), Δ	47	-0.08 ± 1.92	64	-0.84 ± 2.34	0.064
Lateral phoria (Near), Δ	47	-3.64 ± 6.37	64	-6.99 ± 6.96	**0.011**
PFV (Distance) Break, Δ	46	18.22 ± 7.52	60	14.82 ± 5.74	**0.010**
PFV (Near) Blur, Δ	37	12.84 ± 6.20	45	9.49 ± 5.45	**0.011**
PFV (Near) Break, Δ	46	19.61 ± 7.48	62	15.35 ± 7.02	**0.003**
PFV (Near) Recovery, Δ	46	11.1 ± 5.79	62	8.69 ± 6.23	**0.043**

AS = activation score. SD = standard deviation. PD = probability distribution. Δ = prism diopters.

Finally, at the descriptive level, we found differences that were not statistically significant when we compared the subjects with PI greater than or equal to 10 (n = 39 average = -0.03 SD ± 2.08 PD) with those who had PI with less than 10 points (n = 72 average = -0.79 SD ± 2.22 PD) in the distance of lateral phoria (p = 0.079, effect size (Cohen's d) = 0.35).

### Relationship between variables that define the binocular vision and those that describe the state of the cervical region

When grouping the subjects according to the normative values of the variables that defined the state of binocular vision, we found that, in those who were not within the norm, there was a higher percentage of participants with AS below the norm. In contrast, in subjects without binocular vision impairment, there was a higher percentage with normal AS. Finally, a greater percentage of the subjects with alteration of the Vergence facility (near) suffered neck pain (they had cervical pain of three or more months of evolution) than in the participants with Vergence facility (near) within the norm. These analyses are shown in Table 5.

**Table 5:** Comparison of the percentages of subjects with binocular vision inside and outside the norm according to having an AS 4 points or higher as opposed to less than 4 points.

Variable		AS			Cervicalgia 3 months		
		≥ 4 n (%)	< 4 n (%)	p-value	No n (%)	Yes n (%)	p-value
Sheard´s Criterion (Near)	Satisfy	28 (52.8)	25 (47.2)	**0.046**	-	-	**-**
	Do not satisfy	19 (33.9)	37 (66.1)		-	-	
NFV (Distance) Break, Δ	Inside the norm	42 (45.7)	50 (54.3)	0.074	-	-	-
	Above the norm	4 (22.2)	14 (77.8)		-	-	
PFV (Near) Blur, Δ	Inside the norm	18 (64.3)	10 (35.7)	**0.019**	-	-	**-**
	Below the norm	19 (35.2)	35 (64.8)		-	-	
Vergence facility testing (Near), cpm	Inside the norm	-	-	-	10 (58.8)	7 (41.2)	0.059
	Below the norm	-	-		24 (33.3)	48 (66.7)	

AS = activation score.

## Discussion

In our investigation, a complete evaluation of binocular vision status was proposed, through tests that presented the highest repeatability, to determine the possible existence of nonstrabismic binocular dysfunctions and to analyze whether there was a relationship between the visual system and neck complaints.

The analysis of our variables indicated that subjects with binocular vision impairment showed a decreased response of the deep cervical musculature, with low AS and PI levels. They also showed a tendency to suffer cervicalgia of more than three months of evolution. These results are consistent with previous studies demonstrating the relationship between the visual system and the musculoskeletal system of the neck [7–13].

Several symptoms and signs can be used to diagnose binocular anomalies. However, there is a lack of consensus in scientific literature about which diagnostic criteria should be used to define each dysfunction [4,34,35]. In the present study, we used the same diagnostic criteria as proposed by Jiménez R et al. [17], based on the variables horizontal heterophoria, range of horizontal vergences in both internal and external base directions, range of vertical vergences and vergential flexibility.

Taking into account all the variables that defined binocular vision, essentially two groups were distinguished in our sample: those who presented nonstrabismic binocular dysfunctions (excess divergence, fusional vergence dysfunction (FVD), insufficiency of convergence, vertical dysfunction and unstable binocular vision [34,35]) and the group of participants with normal binocular vision.

We identified subjects who presented values of NFV (distance) break above the norm, a condition that causes a tendency toward greater amplitudes of divergence and is associated with greater exodeviation in far than in near, signs that characterize an excess of divergence [36,37]. This group presented low AS and PI compared to the group whose NFV (distance) break value was normal (Fig 1).

On the other hand (Table 3), we identified subjects with values of NFV (near) break, NFV (near) recovery, PFV (distance) break, VV (distance) recovery and VV (near) recovery out of the norm, which determined altered horizontal and vertical fusion amplitude ranges and which are associated with deviations in far and near [38–40]. In this group of subjects, the extension, right side-bending, and left rotation were diminished.

When we classified subjects according to the state of the cervical region, we detected that participants with AS below normal present diminished values of blur, break and recovery PFV (near), a situation that leads to a tendency to decrease the range fusional convergence amplitude, signs that can be associated with the presence of insufficiency of convergence (Table 4) [38,41–43]. It has also been observed that subjects with NDI values above normal, that is, with cervical disability, present recoveries below normal in the VV for far, a clinical sign that can be associated with vertical dysfunction [44], whereas participants with normal NDI levels had normal recoveries in VV for far.

When analyzing the existing relationships, considering the normative values of both the variables that defined the binocular vision status and the variables referring to the state of the cervical region, we found that in subjects with a smaller amplitude of PFV near, a situation that is identified with insufficiency of convergence [38,41–43], a higher percentage presented altered AS values (below 4 points). Similarly, in the group of subjects who did not comply with Sheard's criterion (near), a situation that is identified with unstable binocular vision, there was a higher percentage of individuals with decreased AS values In the same way, among the subjects who had NFV (distance) break above the norm (excess of divergence) [36,37,45] and subjects who had PFV (near) blur values below the norm (insufficiency of convergence) [38,41–43], we found a higher percentage with decreased levels of AS (below 4). On the other hand, in subjects with decreased vergence facility (near) (fusional vergence dysfunction) [30,46], a greater percentage reported cervical pain of three or more months of evolution and therefore a tendency to suffer cervicalgia compared to subjects in whom the value of vergence facility (near) was normal; among the latter, a lower number reported pain.

Our literature review showed two hypotheses explaining the relationship between binocular dysfunction and neck dysfunction.

### A cervical problem can cause an alteration of binocular vision

Three reflexes influence head, eye and postural stability [47], which depend on cervical afferents: the cervico-colic reflex (CCR), the cervico-ocular reflex (COR) and the tonic neck reflex (TNR). These reflexes carry out their functions together with others, being influenced by vestibular and visual input for coordinated stability of the head, eyes and posture. The COR works with the vestibular-ocular reflex (VOR) and the optokinetic reflex (OKR), acting on the extraocular muscles, to maintain stable vision in the retina during head movement. This reflex responds to proprioceptive signals that come from the deep muscles of the neck and the joint capsules from C1 to C3 to reach the vestibular nuclei [48]. A greater gain in COR has been demonstrated in whiplash patients [48]. In this context, an altered cervico-ocular reflex in subjects with neck pain could modify the tone of the extraocular muscles, leading to the destabilization of a phoria by altering the range fusional vergences and thus appearing a binocular alteration.

### On the other hand, we propose that a binocular dysfunction can cause neck dysfunction

A subject with altered binocular vision has reported various symptoms [4] and a modification in neck posture. This is due to an adaptation of the head, to maintain binocularity and optimize visual acuity, which can cause musculoskeletal problems. Zhang et al. [49] observed an abnormal head posture in a group of children while watching television. The subjects had reduced ranges of horizontal fusional vergence in both directions, convergence and divergence, so they had a small binocular vision area, which was compensated by twisting the head. Maxwell et al. [50,51] and Irsch et al. [44] report how vertical disparity decreases by tilting the head. Nucci et al. [52] suggest that involvement in the neck muscles may be secondary to an inclination of the head while trying to compensate for a vertical deviation due to involvement of the superior oblique muscle. In this way, the head can be placed in a position where reflexively there is a decrease in the tone of the affected eye muscles. This postural adaptation would be good for improving vision but would lead to joint and muscular dysfunctions in the neck, giving rise to a cervical pathology if maintained over time. Seen this way, neck pain would be a trade-off for the improvement of visual acuity. Cervical pathology can be the result of permanent compensation to the service of visual comfort [53].

Given the relationship between both systems, the visual and cervical, future research might propose an intervention consisting of a visual therapy program in subjects with nonstrabismic binocular dysfunction and neck pain, because visual therapy is a useful treatment option in subjects with binocular anomalies [54], and it could assess whether there are changes in possible neck dysfunctions.

## Conclusion

After an evaluation of the complete and exhaustive binocular function, we conclude that there is a relationship between nonstrabismic binocular dysfunction and neck pain. The binocular anomalies detected in the sample under study were excess divergence, insufficiency of convergence, vertical dysfunction and fusional vergence dysfunction, and unstable binocular functions were correlated with low activation score and performance index of the deep neck musculature, less mobility of the neck, greater functional disability, and greater cervical pain.

## Supporting information

S1 File (XLSX)
